# Supplementary material for: The role of arsenic in the operation of sulfur-based electrical threshold switches
Source: Nat Commun. 2023 Sep 29;14:6095. doi: 10.1038/s41467-023-41643-6 (PMC10542328; doi:10.1038/s41467-023-41643-6)
Supplement: Supplementary file 1 — Supplementary Information [file 41467_2023_41643_MOESM1_ESM.pdf]

Supplementary Materials for

The Role of Arsenic in the Operation of Sulfur-based Electrical Threshold Switches

Renjie Wu *et al.*

* Corresponding authors. Email: minzhu@mail.sim.ac.cn

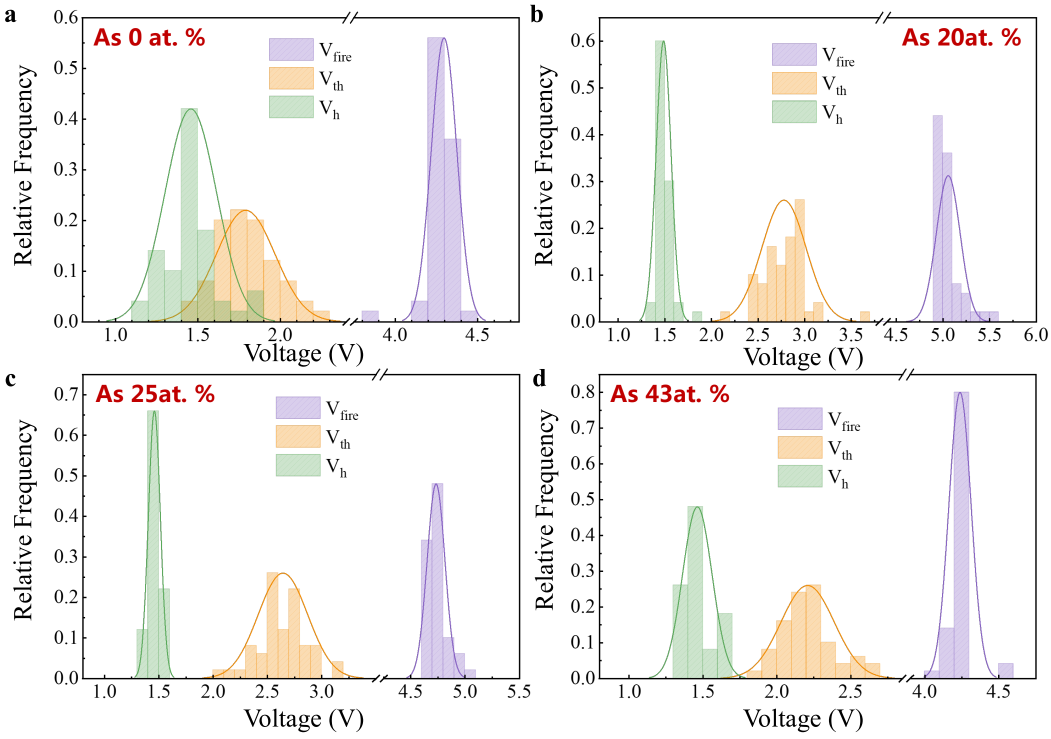

Fig. S1 V_{fire}, V_{th} and V_h distributions of a. GeS, b. $GeSAs_{20}$, c. $GeSAs_{25}$ and d. $GeSAs_{43}$ devices (50 devices for each composition). V_{fire} of GeS, $GeSAs_{25}$ and $GeSAs_{43}$ devices are obtained by the device responses to the 6 V triangular pulses, and V_{fire} of $GeSAs_{20}$ is measured by a 6.5 V one. V_{th}, V_h and I_{on} of devices with different As concentrations are determined by 3 V, 4.5 V, 4 V and 4 V pulses, respectively. The rising and falling edges of all pulses are 1 μs. Moreover, I_{off} is measured through DC test and the step is 0.1 V. **a.** The V_{fire}, V_{th} and V_h values for pure GeS range from 4.1 to 4.5 V, 1.3 V to 2.3 V and 1.1 V to 1.9 V, respectively. The window between V_{th} and V_h might result in errors in the read process in memory applications. With the addition of As, V_h stabilizes at about 1.5 V, and the fluctuation of V_{th} values diminished. **b.** The V_{fire}, V_{th} and V_h values for $GeSAs_{20}$ range from 4.9 to 5.6 V, 2.4 V to 3.1 V and 1.3 V to 1.7 V, respectively. **c.** The V_{fire}, V_{th} and V_h values for $GeSAs_{25}$ range from 4.6 to 5 V, 2 V to 3 V and 1.3 V to 1.6 V, respectively. **d.** The V_{fire}, V_{th}, and V_h values for $GeSAs_{43}$ range from 4 to 4.6 V, 1.8 V to 2.7 V, and 1.3 V to 1.7 V, respectively.

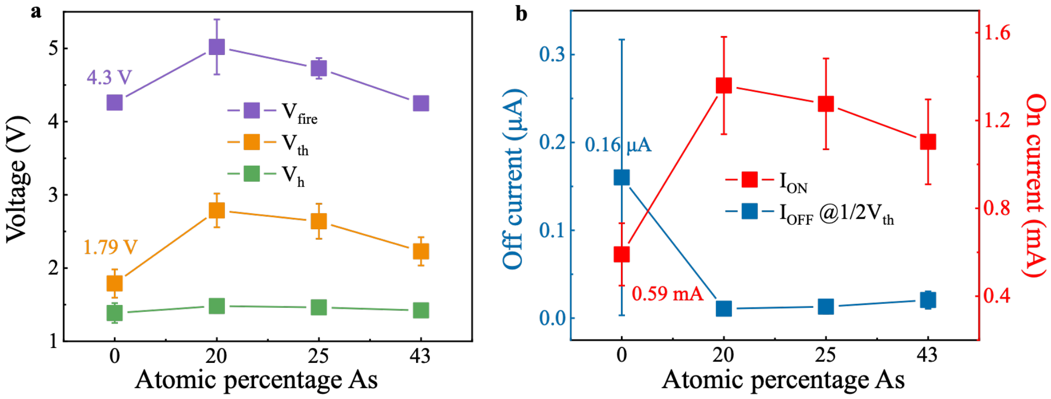

Fig. S2 Horizontal comparation of voltage and current parameters of GeS, $GeSAs_{20}$, $GeSAs_{25}$ and $GeSAs_{43}$ devices. a. The fluctuation of V_{fire}, V_{th} and V_{h} of 50 individual devices. V_{fire} of GeS, $GeSAs_{25}$ and $GeSAs_{43}$ devices are obtained by the device responses to the 6 V triangular pulses, and V_{fire} of $GeSAs_{20}$ is measured by a 6.5 V one. V_{th}, V_{h} and I_{on} of devices with different As concentrations are determined by 3 V, 4.5 V, 4 V and 4 V pulses, respectively. The rising and falling edges of all pulses are 1 μs. The regulation of voltage seems to be similar with that of the I_{on}. V_{fire} goes up from 4.3 V to 5 V, goes down to 4.7 V next, and ends up at 4.4 V, while V_{th} jumps from 1.79 V to 2.79 V and decreases to 2.64 V and then to 2.23 V. V_{h} basically remained unchanged at 1.5 V. **b.** The variation of I_{on} and I_{off} with As content. I_{on} was captured at the point of threshold switching. Moreover, I_{off} is measured through DC test and the step is 0.1 V. I_{off} refers to the current at 1/2 V_{th} in the subthreshold region. In concert with the growing trend of As, the mean value of I_{off} from 30 devices for each As content starts at 0.16 μA at 1 V, then goes down to 0.11 nA at 1.5 V, which is the turning point, then slowly climbed to 0.13 nA at 1.5 V, and it kept increasing to 0.2 nA at 1.2 V finally. It can be seen from the figure that the addition of As immensely improved the uniformity of I_{off}. The ON state current goes up from 0.59 mA to 1.36 mA and then down to 1.11 mA.

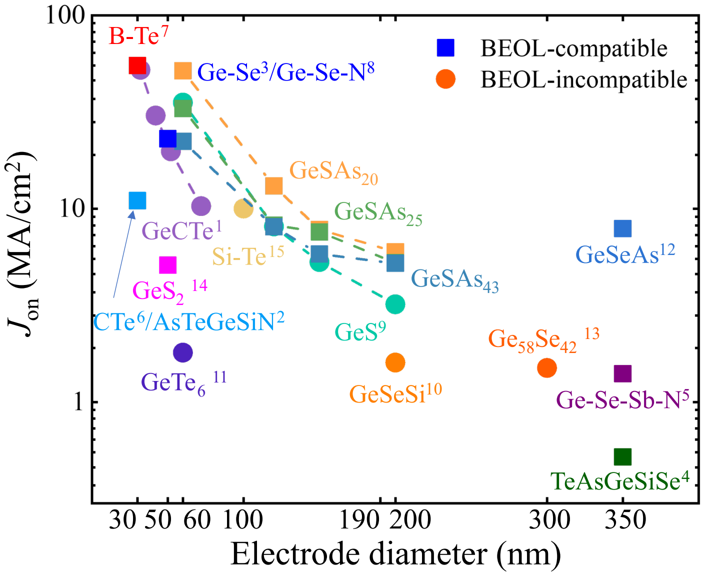

Fig. S3 Comparison of the on-current density of GeS, $GeSAs_{20}$, $GeSAs_{25}$ and $GeSAs_{43}$ devices with that of other OTS cells. The on-current density (J_{on}) quadratically increases with a decrease of the device size. GeSAs shows advantages in terms of current density as well as thermal stability[1–15].

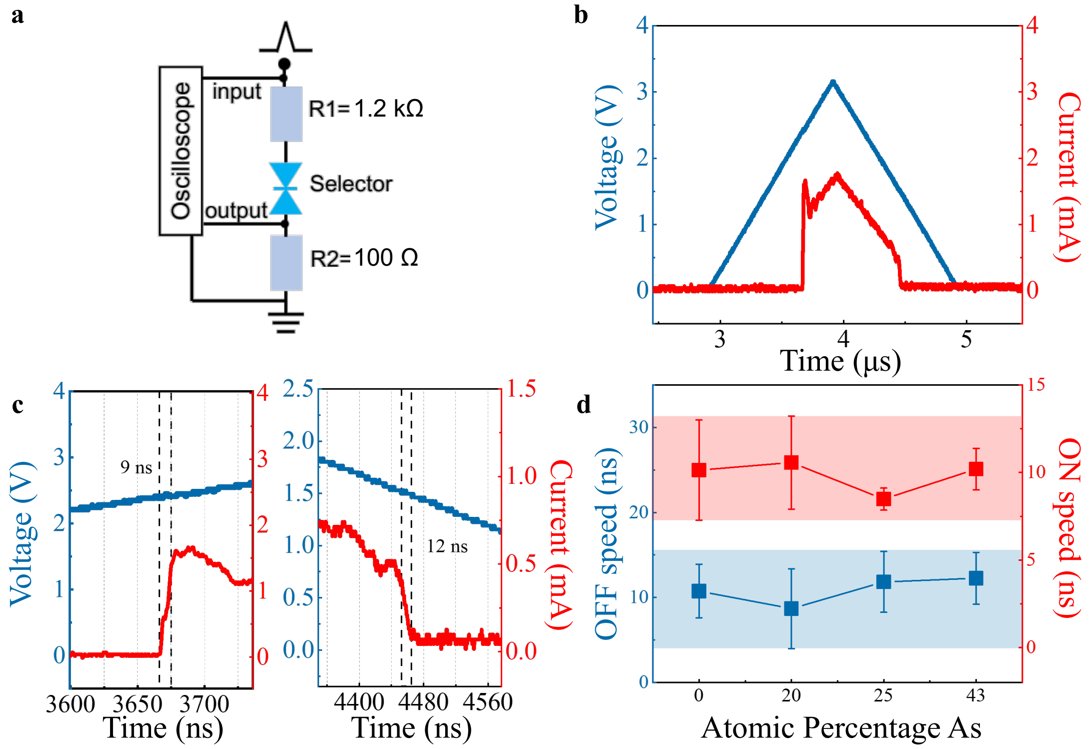

Fig. S4 On and off speeds of GeS, $GeSAs_{20}$, $GeSAs_{25}$ and $GeSAs_{43}$ devices. a. To measure the electrical characteristics, a 1.2 kΩ and a 100 Ω resistor were connected in series with GeSAs devices. The 1.2 kΩ resistor is used to limit the current, and the oscilloscope collects the voltage signal across the 100 Ω one. **b.** Integration of input voltage and output current. The input voltage is a triangular pulse with a rising time of 1 μs. The output voltage signal is captured by the oscilloscope and the result is converted into current, as shown in the figure, from which an obvious switching phenomenon can be observed. **c.** Enlarged views of the current response of on- and off-states. When the input voltage reaches V_{th}, the current signal suddenly surges. With a further increase of the input voltage, the upward trend slows down. We interpret the steep increase period as the on-speed. Similarly, the sudden drop of current is taken as the off-speed **d.** Speed statistics of 30 individual devices of all compositions. The on-speed of devices with different compositions ranges from 7 to 12 ns, and the off speed is in the range of 5~15 ns.

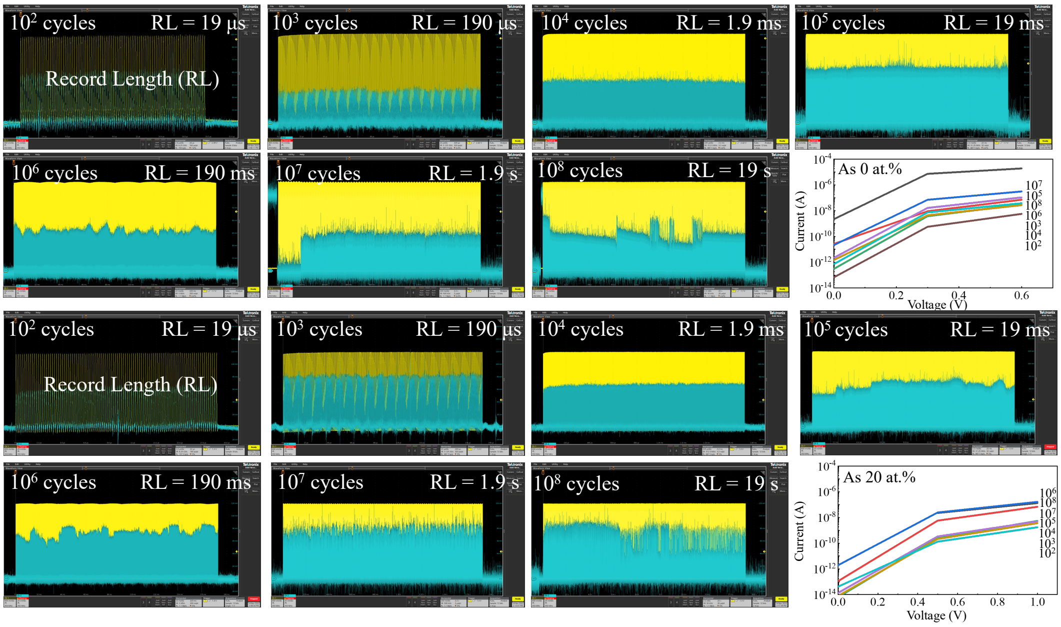

Fig. S5 Endurance of unannealed GeS and $GeSAs_{20}$ devices. 2.5 V and 3.5 V square pulses with 20 ns rising and falling edges, 100 ns pulse width and 50 ns interval were used in the endurance measurements, respectively. The dynamical responses of the switches were measured for a set number of square pulses. Yellow curves correspond to the applied voltage pulses, while the blue curves correspond to the dynamical response measured across the resistor in series with the device. The on-current could be obtained from the dynamical-response curves. Clearly, the GeS and $GeSAs_{20}$ devices were successfully turned on and off by each pulse. After the set number of square pulses operation, the DC *I-V* curves of these devices were obtained, from which off-currents were obtained, as summarized in **Fig. 1**f. The GeS and $GeSAs_{20}$ devices cells work well up to 10^8-cycle pulse operations in this case.

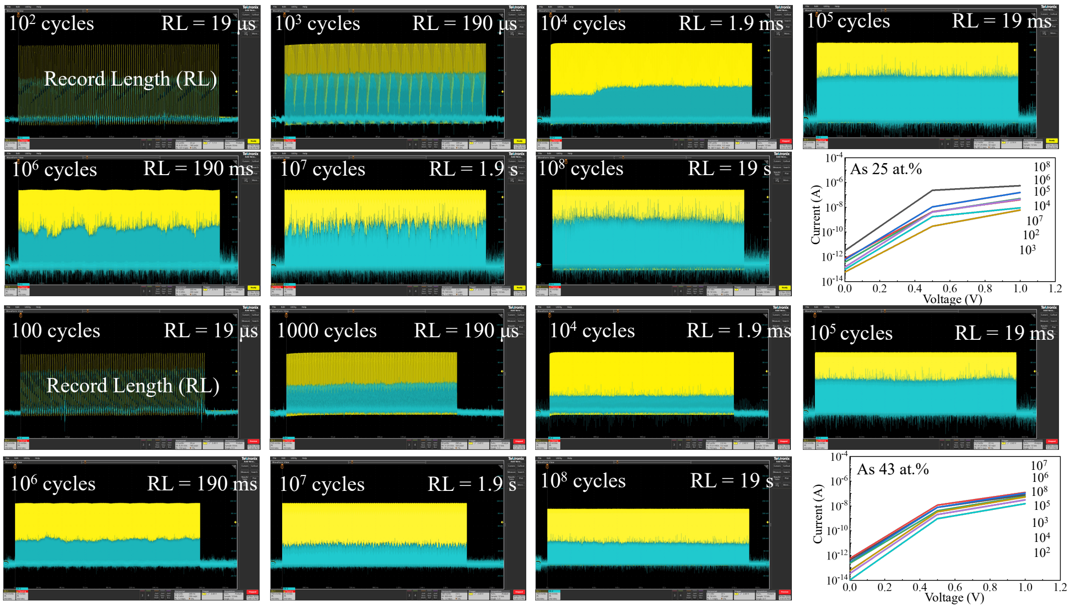

Fig. S6 Endurance of unannealed $GeSAs_{25}$ and $GeSAs_{43}$ devices. 3.5 V square pulses with 20 ns rising and falling edges, 100 ns pulse width and 50 ns interval were used in the endurance measurements. $GeSAs_{25}$ and $GeSAs_{43}$ devices were successfully turned on and off for 10^8 cycles, as shown in **Fig. 1**f.

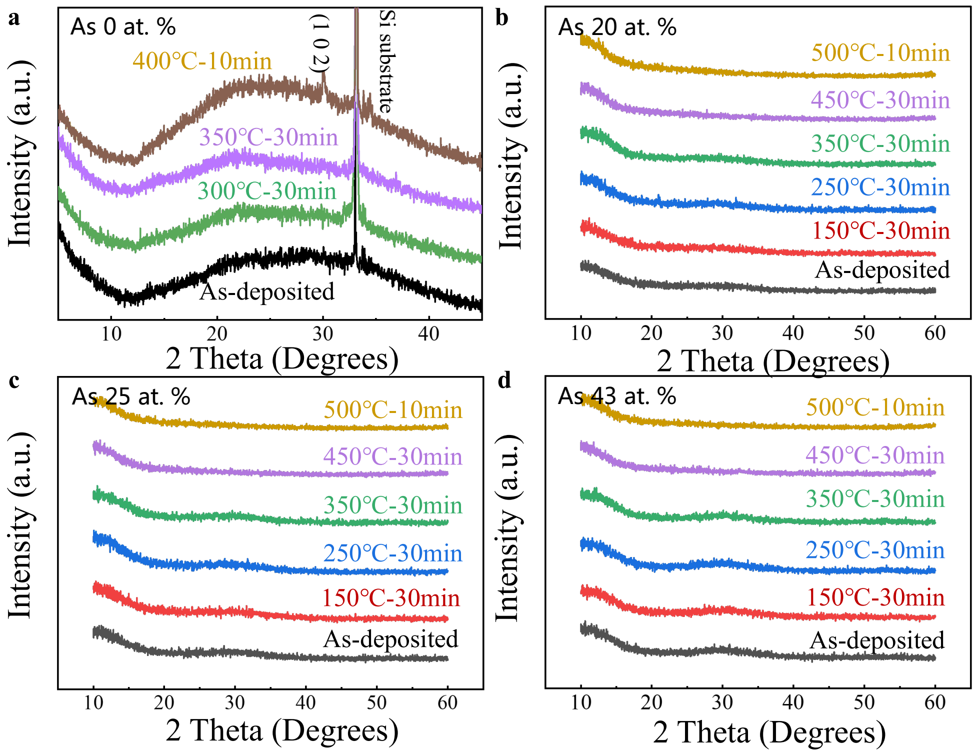

Fig. S7 X-ray diffraction (XRD) results of a. GeS, b. $GeSAs_{20}$, c. $GeSAs_{25}$ and d. $GeSAs_{43}$ films annealed at different temperatures. a. XRD results of GeS film samples annealed at 300 °C and 350 °C for 30 minutes and 400 °C for 10 minutes. Clearly, there is a very small crystallization peak at 30° in the GeS sample annealed at 400 °C, indicating that the crystallization temperature of amorphous GeS is between 350 °C and 400 °C. **b-d.** XRD results of GeSAs samples processed at 150, 250, 350 and 450 °C for 30 minutes and at 500 °C for 10 minutes. The XRD results of the annealed GeSAs samples are almost the same as that of the as-deposited samples, which confirms that As incorporation has a positive effect on the thermal stability of a-GeS.

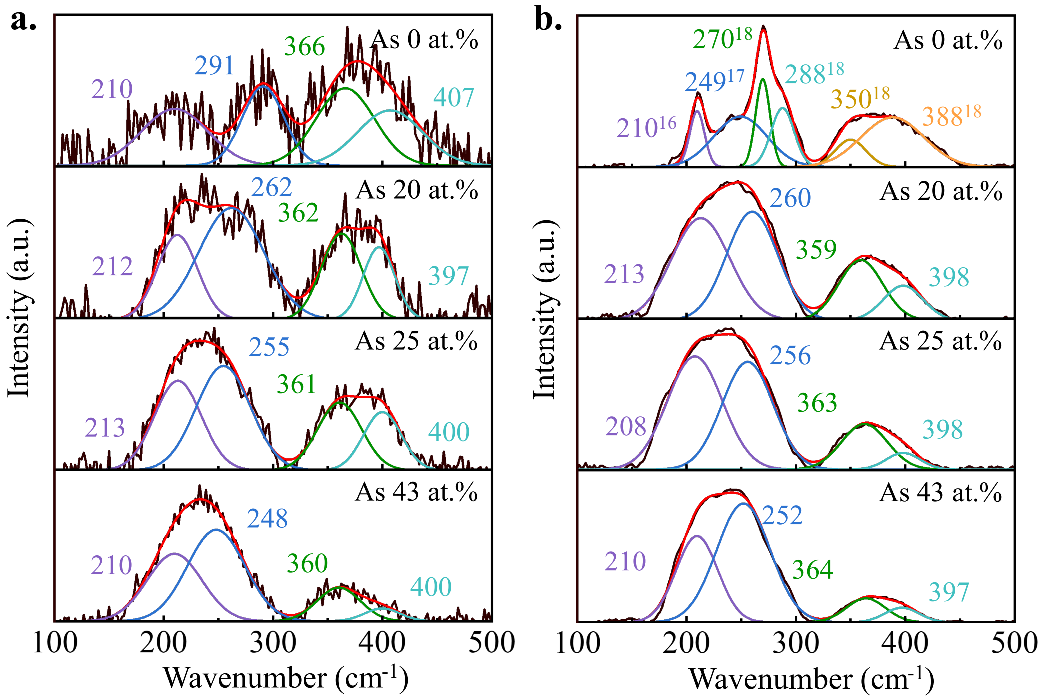

Fig. S8 Raman of GeSAs films. a. Raman spectra of as-deposited GeSAs films. The specific peak fitting results are shown in **Fig. 4**e. **b.** Raman spectra of annealed GeSAs films. GeS film is annealed at 400 °C for 10 minutes and others are annealed under 450 °C for 30 minutes. Then the Raman spectra of annealed films are obtained by LabRAM HR800 Raman spectrometer. It can be observed that there are almost no changes in the peak positions of $GeSAs_{20}$, $GeSAs_{25}$, and $GeSAs_{43}$ after annealing in **Fig. S**8b. However, GeS shows significant differences after annealing with peaks at 210 and 249 cm^{-1} representing the B_{2g} vibration mode[16] and bond bonding motions of S atoms in GeS_4 tetrahedra which appear in crystal phase[17]. Also, GeS still contains amorphous part at this time with peak at 270 cm^{-1} corresponding to SGe_3-$S_{6/3}$ groups[18], peak at 288 cm^{-1} corresponding to SGe_3 pyramids[18], peak at 350 cm^{-1} corresponding to edge-shared GeS_4[18], and peak at 388 cm^{-1} corresponding to S_3Ge-GeS_3 units[18]. Hence, we conclude that GeS crystallizes at 400 °C, and GeSAs materials enable to withstand thermal shock at 450 °C, which is consistent with XRD.

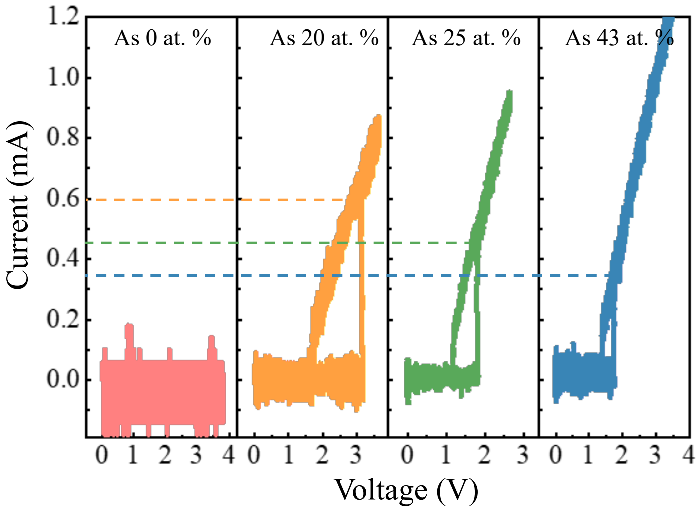

Fig. S9 *I-V* curves of GeSAs devices with 60-nm electrodes after annealing at 450 °C for 30 minutes. The testing pulses are the same as 200-nm as-deposited GeSAs devices. The devices with As involved can all be turned on normally. The on-current of $GeSAs_{20}$, $GeSAs_{25}$ and $GeSAs_{43}$ are 0.6 mA, 0.45 mA and 0.33 mA, respectively.

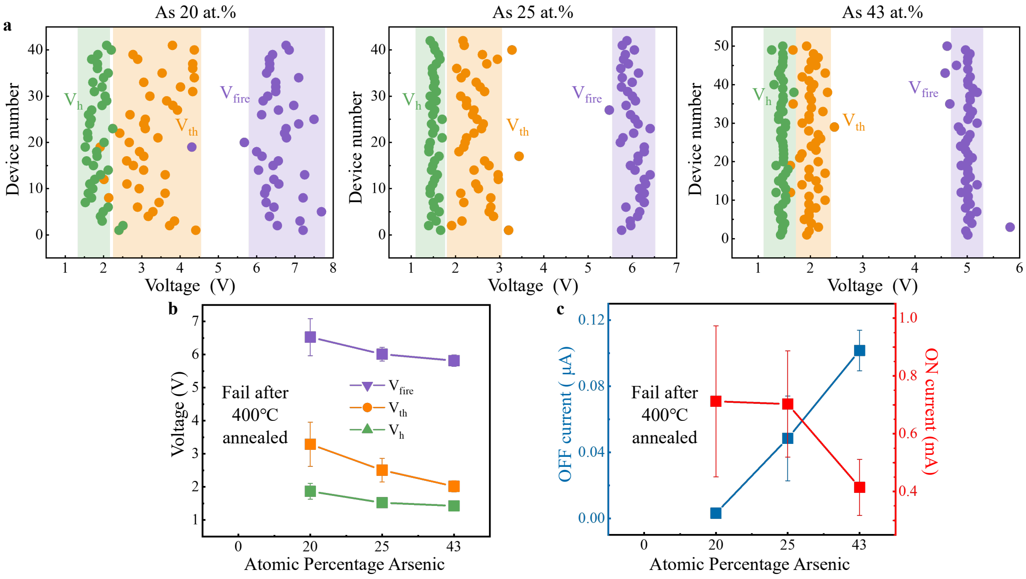

Fig. S10 Distributions and change trends of operation voltages for annealed $GeSAs_{20}$, $GeSAs_{25}$ and $GeSAs_{43}$ cells. a. V_{fire}, V_{th} and V_h distributions for 10 nm thick, 450°C annealed devices containing As. GeS devices failed after 400 °C annealing. V_{fire} of $GeSAs_{20}$, $GeSAs_{25}$ and $GeSAs_{43}$ devices are obtained by the device responses to the 8.5 V, 7 V and 7 V triangular pulses. V_{th}, V_h and I_{on} of devices with different As concentrations are determined by 5 V, 4 V and 4 V pulses, respectively. The rising and falling edges of all pulses are 1 μs. Moreover, I_{off} is measured through DC test and the step is 0.1 V. **b.** After annealing at 450 °C for 30 min, GeS loses its switching performance, while the compositions containing As still work. By increasing the As content, the tendency of changes of V_{fire}, V_{th} and V_h is consistent with that before annealing, and V_{fire} decreases from 6.5 V to 6.2 V and then to 6 V; V_{th} decreases from 3.3V to 2.5 V and then to 2 V. **c.** The device leakage current increases from 3.1×10^{-9} A to 1×10^{-7} A with increasing As content. The on-current of the devices decreases from 0.71 mA to 0.4 mA, which is also consistent with the trend before annealing.

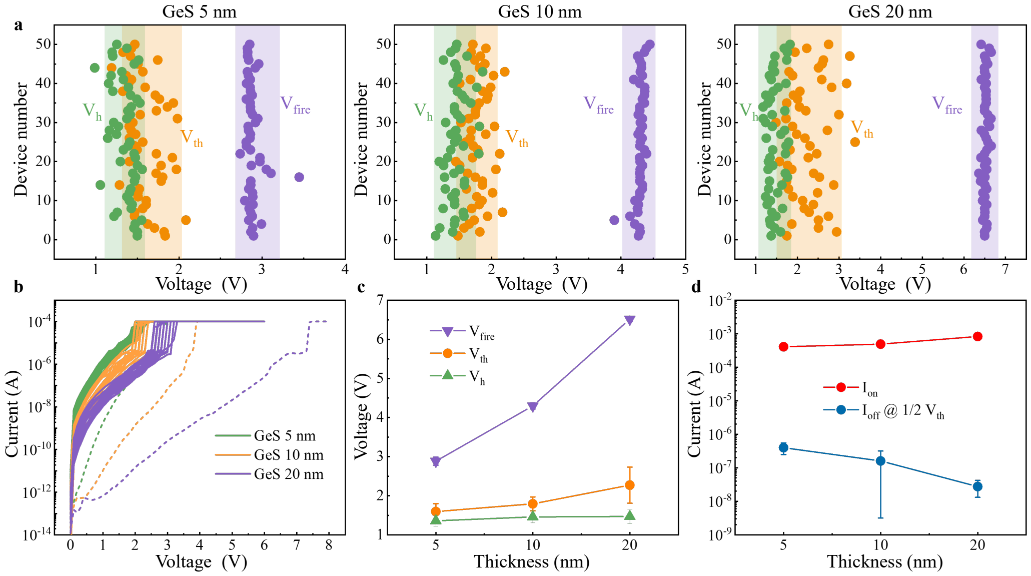

Fig. S11 Distributions and change trends of operation voltages and currents for GeS devices with different thicknesses. a. V_{fire}, V_{th} and V_{h} distributions for as-deposited GeS devices with 5, 10 and 20 nm thick OTS layers. V_{fire} of 5-nm, 10-nm and 20-nm GeS devices are obtained by the device responses to the 4.5 V, 6 V and 8 V triangular pulses. V_{th}, V_{h} and I_{on} of devices with different As concentrations are determined by 2.5 V, 3 V and 4 V pulses, respectively. The rising and falling edges of all pulses are 1 μs. Moreover, I_{off} is measured through DC test and the step is 0.1 V. **b.** I_{off} of deposited GeS devices with 5, 10 and 20 nm thick OTS layer. Dashed lines correspond to the FF process. Solid lines correspond to I_{off}. **c**. V_{fire}, V_{th} and V_{h} variations with thickness. When the As content is zero, V_{fire} rises from 2.8 V, and V_{th} rises from 1.75V, which increase nonlinearly with thickness, and V_{h} is independent of thickness. **d**. I_{on} and I_{off} variations with thickness. I_{on} increases and I_{off} decreases with increasing thickness.

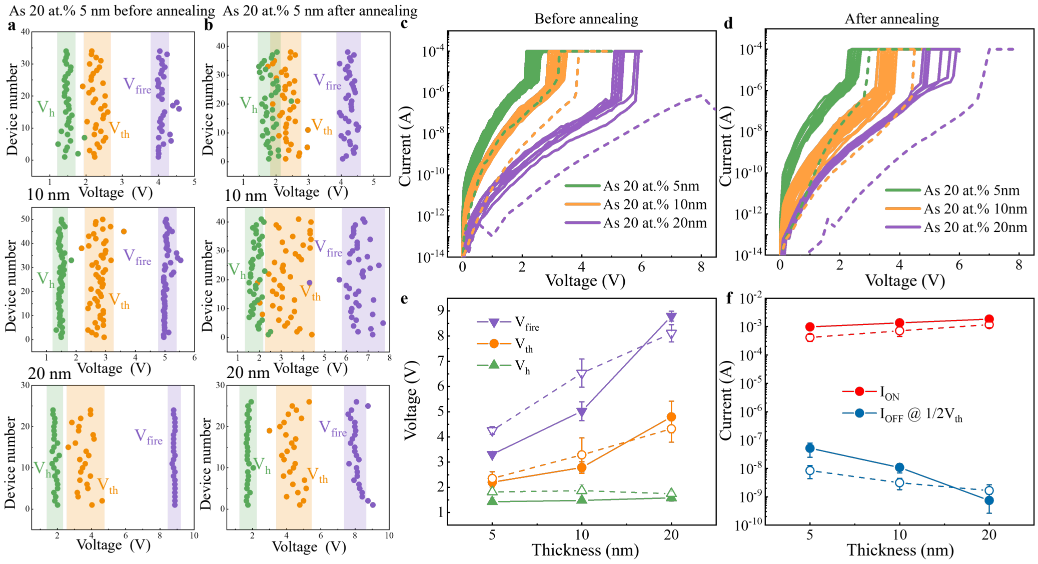

Fig. S12 Comparisons of operation voltages and currents of $GeSAs_{20}$ before and after annealing. a. V_{fire}, V_{th} and V_h distributions for as-deposited $GeSAs_{20}$ devices with 5, 10 and 20 nm-thick OTS layers. **b.** V_{fire}, V_{th} and V_h distributions for annealed $GeSAs_{20}$ devices with 5, 10 and 20 nm thick OTS layers. In 5-nm cells, the V_{fire}, V_{th} and V_h values are distributed from 3.95 V to 4.53 V, 2.12 to 2.72 V, and 1.48 V to 2.06 V, respectively. In 10-nm cells, the V_{fire}, V_{th} and V_h values are distributed from 6 V to 7.69 V, 2.42 to 4.37 V, and 1.52 V to 2.08 V, respectively. In 20-nm cells, the V_{fire}, V_{th} and V_h values are distributed from 7.6 V to 8.4 V, 3.61~5.32 V, and 1.64 V to 2.05 V, respectively. **c.** I_{off} of as-deposited $GeSAs_{20}$ devices with 5, 10 and 20 nm thick OTS layers. Dashed lines correspond to the FF process. Solid lines correspond to I_{off}. **d.** I_{off} of annealed $GeSAs_{20}$ devices with 5, 10 and 20 nm thick OTS layers. Dashed lines correspond to the FF process. Solid lines correspond to I_{off}. **e.** V_{fire}, V_{th} and V_h variations, before and after annealing, with thickness. Solid lines correspond to the as-deposited state. V_{fire} increases from 3.3 V to 5 V and then to 8.8 V with a doubling of the thickness. V_{th} increases from 2.2 V for 5 nm, to 2.8 V for 10 nm and ends up at 4.8 V for 20 nm. V_h remains almost unchanged. Dashed lines correspond to the annealed state. V_{fire} increases from 4.26 V to 6.53 V and then to 8.11 V with a doubling of the thickness. V_{th} increases from 2.35 V for 5 nm, to 3.29 V for 10 nm and ends up at 4.32 V for 20 nm. V_h stays nearly the same. **f.** I_{on} and I_{off} variations before and after annealing with thickness. Solid lines correspond to the as-deposited state. I_{on} changes from 1.45 mA to 1.28 mA, and then to 2.3 mA. I_{off} decreases from 51.6 nA to 10.8 nA, and then to 0.74 nA. Dashed lines correspond to the annealed state. I_{on} becomes slightly smaller than those of unannealed cells. I_{on} increases from 0.42 mA to 0.71 mA, and then to 1.17 mA. While I_{off} of the annealed state is lower, it decreases from 8.4 nA to 3.1 nA, and to 1.65 nA.

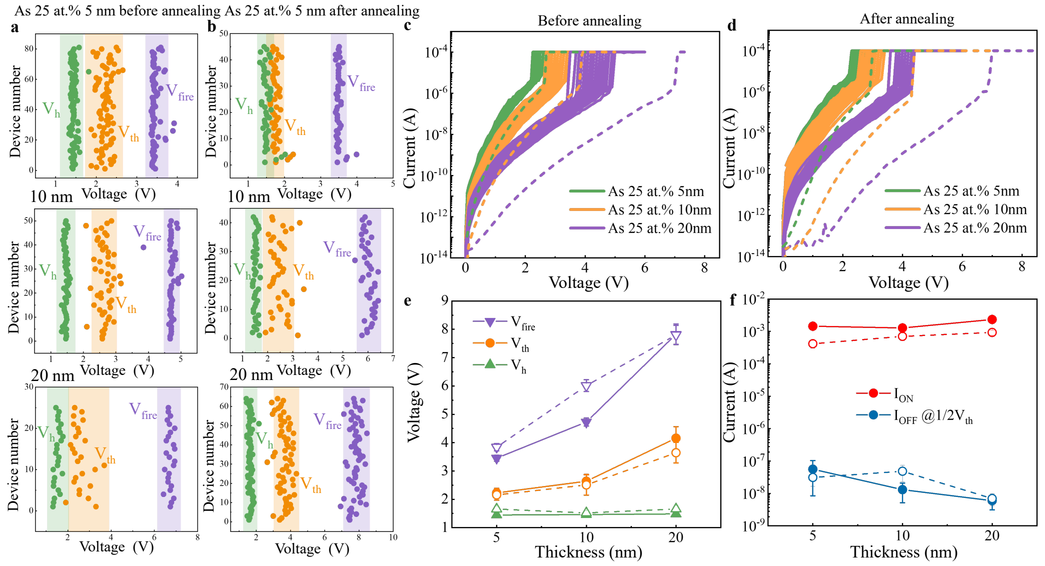

Fig. S13 Comparisons of operation voltages and currents of $GeSAs_{25}$ before and after annealing. a. V_{fire}, V_{th} and V_h distributions for as-deposited $GeSAs_{25}$ devices with 5, 10 and 20 nm thick OTS layers. **b.** V_{fire}, V_{th} and V_h distributions of annealed $GeSAs_{20}$ devices with 5, 10 and 20 nm thick OTS layers. In 5-nm cells, the V_{fire}, V_{th} and V_h values are distributed from 3.41 V to 3.61 V, 1.61 to 1.92 V, and 1.32 V to 1.62 V, respectively. In 10-nm cells, The V_{fire}, V_{th} and V_h values range from 5.76 V to 6.4 V, 1.92 to 2.97 V and 1.4 V to 1.7 V, respectively. In 20-nm cells, The V_{fire}, V_{th} and V_h values range from 6.93 V to 8.47 V, 2.91 to 4.35 V, and 1.42 V to 1.84 V, respectively. **c.** I_{off} of as-deposited $GeSAs_{20}$ devices with 5, 10 and 20 nm thick OTS layers. Dashed lines correspond to the FF process. Solid lines correspond to I_{off}. **d.** I_{off} of annealed $GeSAs_{20}$ devices with 5, 10 and 20 nm thick OTS layers. Dashed lines correspond to the FF process. Solid lines correspond to I_{off}. **e.** V_{fire}, V_{th} and V_h variations before and after annealing with thickness. Solid lines correspond to the as-deposited state. V_{fire} increases from 3.45 V to 4.73 V and then to 7.82 V with a doubling of the thickness. V_{th} increases from 2.23 V for 5 nm, to 2.64 V for 10 nm and ends up at 4.16 V for 20 nm. V_h remains almost unchanged. Dashed lines correspond to the annealed state. V_{fire} increases from 3.84 V to 6.01 V and then to 7.8 V with a doubling of the thickness. V_{th} increases from 2.16 V for 5 nm, to 2.51 V for 10 nm and ends up at 3.65 V for 20 nm. V_h stays nearly the same. **f.** I_{on} and I_{off} variations before and after annealing with thickness. Solid lines correspond to the as-deposited state. I_{on} increases from 1.45 mA to 1.28 mA, and then to 2.3 mA. I_{off} decreases from 56 nA to 13 nA, and then to 0.6 nA. Dashed lines correspond to the annealed state. I_{on} becomes slightly smaller than those of unannealed cells. I_{on} increases from 0.42 mA to 0.7 mA, and then to 0.93 mA. While I_{off} for the annealed state is lower, it changes from 31 nA to 48 nA, and then to 6.9 nA.

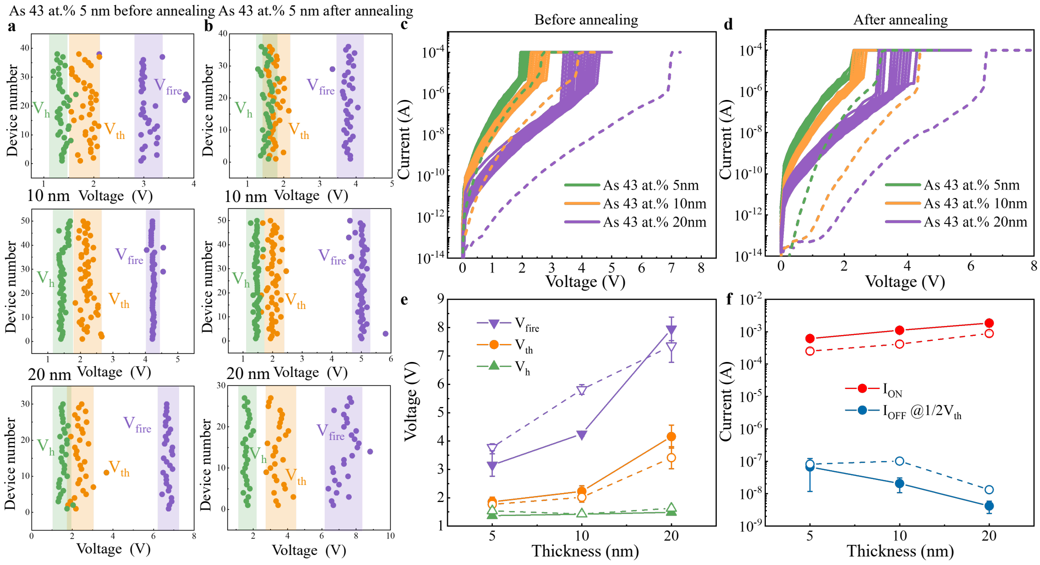

Fig. S14 Comparisons of operation voltages and currents of $GeSAs_{43}$ before and after annealing. a. V_{fire}, V_{th} and V_h distributions of as-deposited $GeSAs_{43}$ devices with 5, 10 and 20 nm thick OTS layers. **b.** V_{fire}, V_{th} and V_h distributions of annealed $GeSAs_{20}$ devices with 5, 10 and 20 nm thick OTS layers. In 5-nm cells, the V_{fire}, V_{th} and V_h values range from 3.63 V to 4.05 V, 1.52 to 2.14 V, and 1.29 V to 1.83 V, respectively. In 10-nm cells, the V_{fire}, V_{th} and V_h values range from 6 V to 7.69 V, 2.42 to 4.37 V, and 1.52 V to 2.08 V, respectively. In 20-nm cells, the V_{fire}, V_{th} and V_h values range from 6.38 V to 8.16 V, 2.76 to 4.34 V, and 1.4 V to 2 V. **c.** I_{off} of as-deposited $GeSAs_{20}$ devices with 5, 10 and 20 nm thick OTS layers. Dashed lines correspond to the FF process. Solid lines correspond to I_{off}. **d.** I_{off} of annealed $GeSAs_{20}$ devices with 5, 10 and 20 nm thick OTS layers. Dashed lines correspond to the FF process. Solid lines correspond to I_{off}. **e.** V_{fire}, V_{th} and V_h variations before and after annealing with thickness. Solid lines correspond to the as-deposited state. V_{fire} increases from 3.15 V to 4.25 V and then to 7.96 V with a doubling of the thickness. V_{th} increases from 1.86 V for 5 nm, to 2.23 V for 10 nm and ends up at 4.16 V for 20 nm. V_h remains almost unchanged. Dashed lines correspond to the annealed state. V_{fire} increases from 3.78 V to 5.82 V and then to 7.36 V with a doubling of the thickness. V_{th} increases from 1.77 V for 5 nm, to 2.02 V for 10 nm and ends up at 3.41 V for 20 nm. V_h stays nearly the same. **f.** I_{on} and I_{off} variations before and after annealing with thickness. Solid lines correspond to the as-deposited state. I_{on} increases from 0.61 mA to 1.1 mA, and then to 1.84 mA. I_{off} decreases from 67.2 nA to 20.6 nA, and to 4.18 nA. Dashed lines correspond to the annealed state. I_{on} becomes slightly smaller than those of unannealed cells. I_{on} increases from 0.25 mA to 0.41 mA, and then to 0.88 mA. While I_{off} of the annealed state is lower, it changes from 80 nA to 0.1 μA, and further to 13.3 nA.

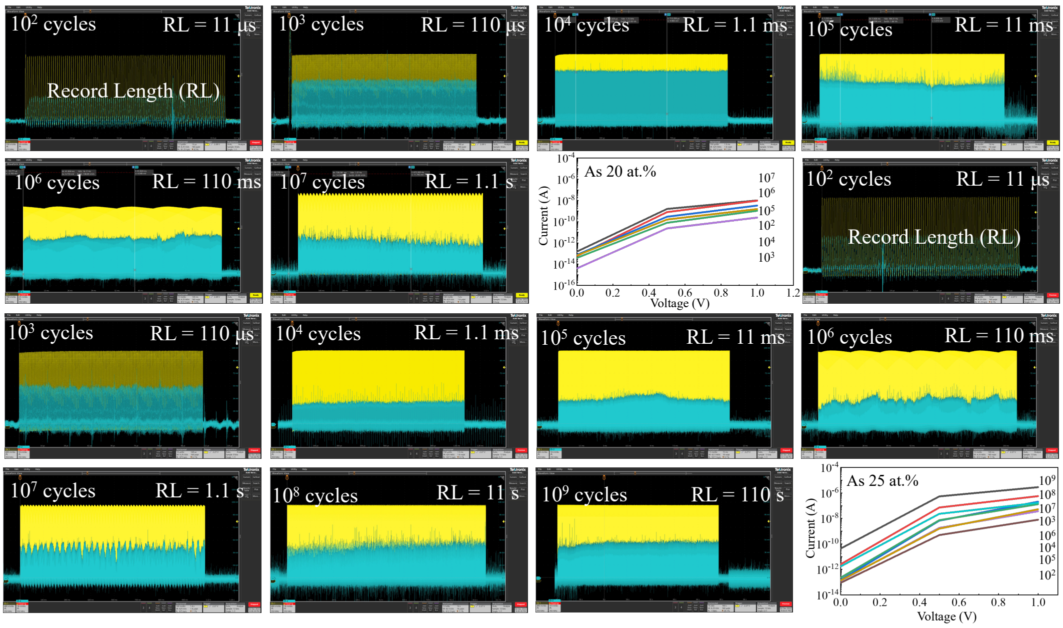

Fig. S15 Endurance of $GeSAs_{20}$ and $GeSAs_{25}$ devices after annealing at 450 °C for 30 min. 4 V square pulses with 20 ns rising and falling edges, 20 ns pulse width and 50 ns interval were used in the endurance measurements. The device endurance of $GeSAs_{20}$ reaches 10^7 cycles and $GeSAs_{25}$ can be operated 10^9 times normally.

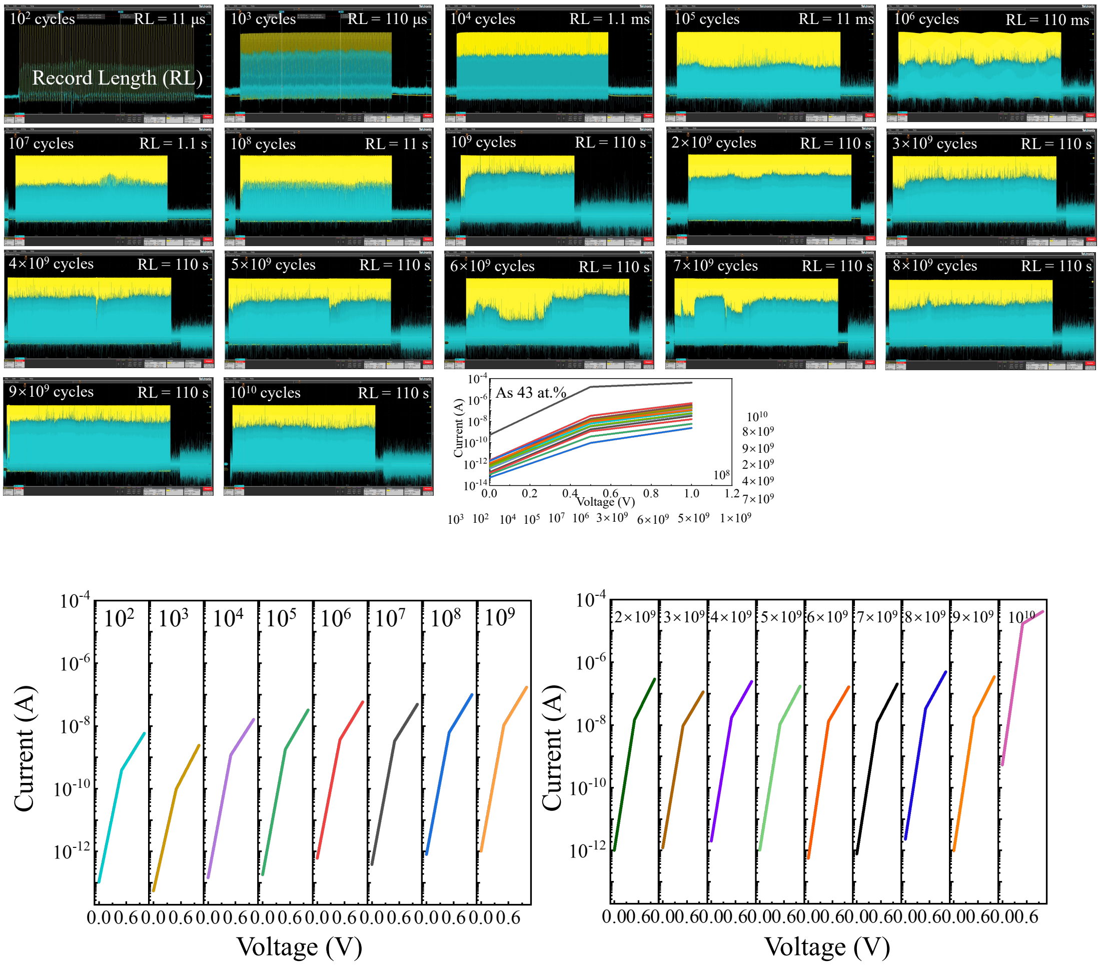

Fig. S16 Endurance of $GeSAs_{43}$ devices after annealing at 450 °C for 30 min. 4 V square pulses with 20 ns rising and falling edges, 20 ns pulse width and 50 ns interval were used in the endurance measurements. The device endurance of $GeSAs_{43}$ reaches 9×10^9 cycles.

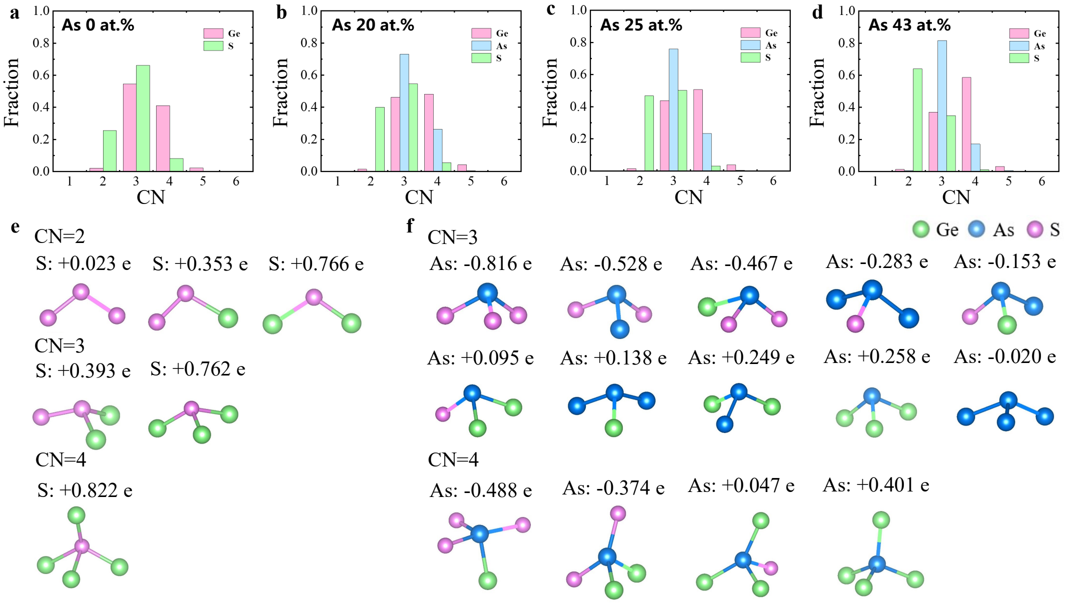

Fig. S17 Correlation between the coordination environment and the charge transfer of central S/As atoms. **a-d.** Coordination numbers (CN) for a-GeS, a-$GeAsS_{20}$, a-$GeAsS_{25}$ and a-$GeAsS_{43}$, respectively. The CNs of Ge/As atoms are concentrated on 3 and 4, and the central S atoms are mainly 2-fold and 3-fold coordinated. **e.** Various coordination environments of central S atoms with the corresponding values of electron transfer. **f.** Various coordination environments of central As atoms with the corresponding electron-transfer values.

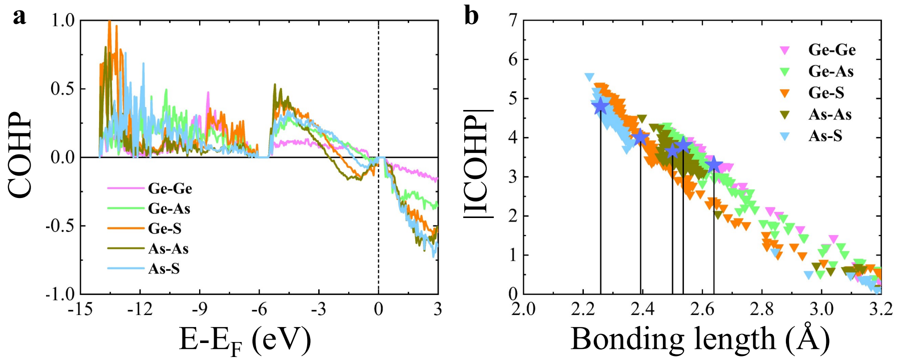

Fig. S18 Analysis of bonding properties for the a-GeSAs$_{43}$ system. a. COHP curves for different bonds in a-GeAsS$_{43}$. **b.** The |ICOHP| curves for different bonds with respect to the bond length in a-GeAsS$_{43}$; the blue stars correspond to the interatomic distances for the first peaks of the PDF, $g(r)$, in **Fig. 3**d, which represents the bond distributions.

The crystal orbital Hamilton populations (COHP) were calculated by the LOBSTER code. **Fig. S**18a shows the COHP curves for different bonds in the model of a-GeAsS$_{43}$, in which the COHP values of As-As, As-S and Ge-S interactions are negative below the Fermi level, indicating that those bonds have antibonding-like character. The COHP for As-S is generally larger than for Ge-S, suggesting that As-S bonds are stronger than Ge-S bonds. To describe the strength of chemical bonds more precisely, one can integrate the COHP (i.e. |ICOHP|) below the Fermi level. The bond strengths marked with the blue stars correspond to the interatomic distances of the first peaks of the PDF, $g(r)$, in **Fig. 3**d. The bond-formation strengths of Ge-Ge, Ge-S, Ge-As, As-As and As-S bonds are calculated to be 3.4 eV, 4 eV, 3.8 eV, 3.65 eV and 4.8 eV, respectively. That is to say, As-S bonds are stronger than Ge-S bonds, thus accounting for the >100 °C increase in the crystallization temperature upon As incorporation.

a

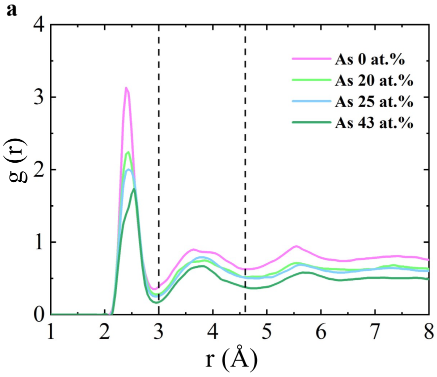

b

Statistics of the second nearest neighbor atoms of Ge in different systems				
	As 0 at. %	As 20 at. %	As 25 at. %	As 43 at. %
Ge-Ge	41.8%	30.7%	27.3%	20.3%
Ge-S	58.2%	44.1%	42.2%	30.7%
Ge-As	—	25.2%	30.5%	49.0%

Fig. S19 The partial PDF and local structure information of a-GeS, a-GeSAs$_{20}$, a-GeSAs$_{25}$ and a-GeSAs$_{43}$ systems. a. The partial PDF, $g(r)$ centered on Ge for a-GeS, a-GeSAs$_{20}$, a-GeSAs$_{25}$ and a-GeSAs$_{43}$ systems. The first peaks and the second peaks represent the separation between nearest-neighbor atoms and between second nearest-neighbor atoms, respectively, from which one can identify that the second nearest-neighbor atomic separation lies between 3.0 and 4.6 Å (dashed lines). The second nearest-neighbor coordination number of Ge atoms can be obtained by integrating $g(r)$ of Ge-Ge, Ge-S and Ge-As correlations between these distances in Fig 3d. **b.** Statistics of the second nearest-neighbor atoms of Ge in different systems. For the second nearest-neighbor atoms of Ge, the fractions of Ge-Ge and Ge-S bonds decreases and the fraction of Ge-As bonds increases.

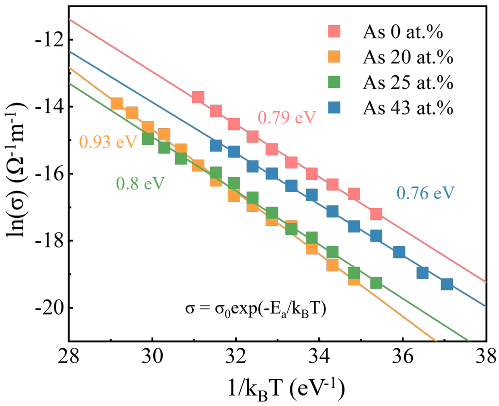

Fig. S20 The electrical conductivity activation energy of a-GeS, a-GeSAs$_{20}$, a-GeSAs$_{25}$ and a-GeSAs$_{43}$ films. The electrical conductivity activation energy can be drawn from the Arrhenius equation: $\sigma = \sigma_0 \exp(-E_a/k_B T)$. E_a is the activation energy for electrical conduction. k_B is the Boltzmann constant and T is the absolute temperature. The value increases from 0.79 to 0.93 eV, then drops to 0.8 eV, and ends up at 0.76 eV with increasing As content, which is consistent with the variations of I_{off} and the bandgap.

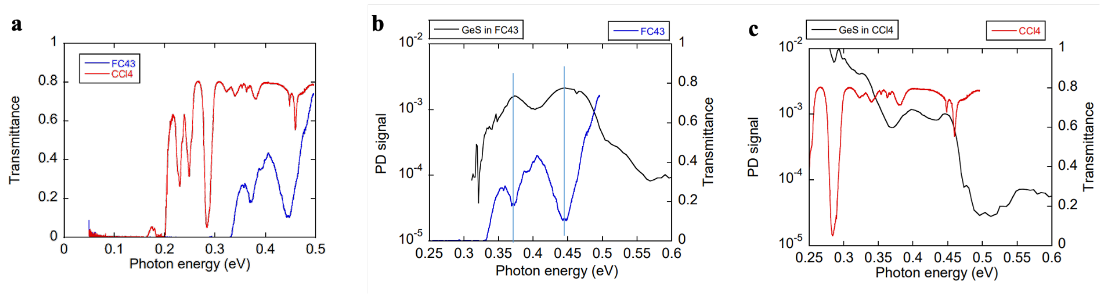

Fig. S21 FTIR and PDS results of GeS films measured in different environments. **a.** Fluorinert FC43 was used for a previous PDS experiment[19], whereas carbon tetrachloride was used in this work. FTIR results for FC43 and carbon tetrachloride for an optical path length of 10 mm are shown. The PDS in this region is subject to the absorption of the deflection medium. Below 0.33 eV, FC43 does not transmit light. There is a region where the transmittance is low, even at 0.33 eV or more. Carbon tetrachloride, on the other hand, has a transmittance of about 80% in most regions above 0.3 eV. **b.** As shown by the vertical lines, there is a good agreement between the absorption position of FC43 and the peak position of the PDS spectrum. **c.** It can be seen that carbon tetrachloride has almost no effect in the high-transmittance region. Absorption around 0.29 eV for carbon tetrachloride affects the spectrum. Therefore, it is concluded that the absorption of the deflection medium is superimposed on the PDS spectrum in the region where the transmittance of the deflection medium is 20% or less. From these results, carbon tetrachloride is suitable for evaluating the trap levels of GeSAs.

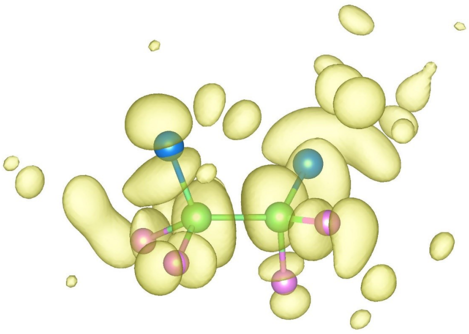

Fig. S22 The atomic configuration corresponding to the gap state near the conduction-band edge with a large IPR value in a-GeSAs20. In the configuration, Ge-Ge bonds also exist but they obey the "8-N" rule, unlike the trap states.

Supplementary References

1. Avasarala, N. S. *et al.* Half-threshold bias Ioff reduction down to nA range of thermally and electrically stable high-performance integrated OTS selector, obtained by Se enrichment and N-doping of thin GeSe layers. in *2018 IEEE Symposium on VLSI Technology* vols 2018-June 209–210 (IEEE, 2018).
2. Ambrosi, E. *et al.* Low variability high endurance and low voltage arsenic-free selectors based on GeCTe. in *2021 IEEE International Electron Devices Meeting (IEDM)* 28.5.1-28.5.4 (IEEE, 2021).
3. Govoreanu, B. *et al.* Thermally stable integrated Se-based OTS selectors with >20 MA/cm 2 current drive, >3.10 3 half-bias nonlinearity, tunable threshold voltage and excellent endurance. in *2017 Symposium on VLSI Technology* vol. 21 T92–T93 (IEEE, 2017).
4. Cheng, H. Y. *et al.* An ultra high endurance and thermally stable selector based on TeAsGeSiSe chalcogenides compatible with BEOL IC Integration for cross-point PCM. in *2017 IEEE International Electron Devices Meeting (IEDM)* vol. 1 2.2.1-2.2.4 (IEEE, 2017).
5. Verdy, A. *et al.* High Temperature Stability and Performance Analysis of N-doped Ge-Se-Sb Based OTS Selector Devices. in *2018 IEEE International Memory Workshop (IMW)* 1–4 (IEEE, 2018).
6. Chekol, S. A. *et al.* A C-Te-based binary OTS device exhibiting excellent performance and high thermal stability for selector application. *Nanotechnology* **29**, 345202 (2018).
7. Yoo, J., Lee, D., Park, J., Song, J. & Hwang, H. Steep Slope Field-Effect Transistors with B-Te-Based Ovonic Threshold Switch Device. *IEEE J. Electron Devices Soc.* **6**, 821–824 (2018).
8. Kim, S. *et al.* Performance of threshold switching in chalcogenide glass for 3D stackable selector. in *2013 Symposium on VLSI Technology* T240–T241 (IEEE, 2013).
9. Jia, S., Li, H., Liu, Q., Song, Z. & Zhu, M. Scalability of Sulfur-Based Ovonic Threshold Selectors for 3D Stackable Memory Applications. *Phys. status solidi – Rapid Res. Lett.* **15**, 2100084 (2021).
10. Li, X., Yuan, Z., Lv, S., Song, S. & Song, Z. Extended endurance performance and reduced threshold voltage by doping Si in GeSe-based ovonic threshold switching selectors. *Thin Solid Films* **734**, 1–9 (2021).
11. Anbarasu, M., Wimmer, M., Bruns, G., Salinga, M. & Wuttig, M. Nanosecond threshold switching of GeTe 6 cells and their potential as selector devices. *Appl. Phys. Lett.* **100**, 143505 (2012).
12. Cheng, H. Y. *et al.* Ultra-High Endurance and Low I<inf>OFF</inf> Selector based on AsSeGe Chalcogenides for Wide Memory Window 3D Stackable Crosspoint Memory. in *2018 IEEE International Electron Devices Meeting (IEDM)* 37.3.1-37.3.4 (IEEE, 2018).
13. Cyrille, M. C. *et al.* OTS selector devices: Material engineering for switching performance. in *2018 International Conference on IC Design & Technology*

(ICICDT) 113–116 (IEEE, 2018).

14. Kim, M. *et al.* PE-ALD of Ge 1−x S x amorphous chalcogenide alloys for OTS applications. *J. Mater. Chem. C* **9**, 6006–6013 (2021).
15. Koo, Y., Lee, S., Park, S., Yang, M. & Hwang, H. Simple Binary Ovonic Threshold Switching Material SiTe and Its Excellent Selector Performance for High-Density Memory Array Application. *IEEE Electron Device Lett.* **38**, 568–571 (2017).
16. Gregora, I. & Stetter, W. Raman Spectra of Crystalline GeS. *Phys. status solidi* **71**, K187–K189 (1975).
17. Kotsalas, I. P. & Raptis, C. High-temperature structural phase transitions of Ge. *Phys. Rev. B* **64**, 125210 (2001).
18. Holomb, R., Johansson, P., Mitsa, V. & Rosola, I. Local structure of technologically modified g-GeS 2 : resonant Raman and absorption edge spectroscopy combined with ab initio calculations. *Philos. Mag.* **85**, 2947–2960 (2005).
19. Jia, S. *et al.* Ultrahigh drive current and large selectivity in GeS selector. *Nat. Commun.* **11**, 4636 (2020).
